# Alarmins of the S100-Family in Juvenile Autoimmune and Auto-Inflammatory Diseases

**DOI:** 10.3389/fimmu.2019.00182

**Published:** 2019-02-15

**Authors:** Dirk Holzinger, Klaus Tenbrock, Johannes Roth

**Affiliations:** ^1^Department of Pediatric Hematology-Oncology, University of Duisburg-Essen, Essen, Germany; ^2^Department of Pediatrics, Medical Faculty, RWTH Aachen, Aachen, Germany; ^3^Institute of Immunology, University of Muenster, Münster, Germany

**Keywords:** S100, alarmins, autoinflammation, biomarker, DAMP, rheumatic diseases

## Abstract

Autoimmune and auto-inflammatory diseases in children are causing chronic inflammation, organ damage, and pain. Although several options for treatment are nowadays available a significant number of patients does not respond sufficiently to current therapies. In these diseases inflammatory processes are triggered by numerous exogenous and endogenous factors. There is now increasing evidence that especially a novel family of pro-inflammatory molecules, named alarmins, play a significant role in inflammatory processes underlying these diseases. Alarmins are endogenous proteins released during stress reactions that confer inflammatory signaling via Pattern Recognition Receptors (PRRs), like the Toll-like receptor 4 (TLR4). The most abundant alarmins in juvenile rheumatic diseases belong to the family of pro-inflammatory calcium-binding S100-proteins. In this review we will give a general introduction in S100-biology. We will demonstrate the functional relevance of these proteins in animal models of autoimmune and auto-inflammatory diseases. We will show the expression patterns of S100-alarmins and correlation to disease activity in different forms of juvenile idiopathic arthritis, auto-inflammatory diseases, and systemic autoimmune disorders. Finally, we will discuss the clinical use of S100-alarmins as biomarkers for diagnosis and monitoring of rheumatic diseases in children and will point out potential future therapeutic approaches targeting inflammatory effects mediated by S100-alarmins.

## Danger Signals, Alarmins and Pattern Recognition Receptors in Inflammation

**Figure 1:**
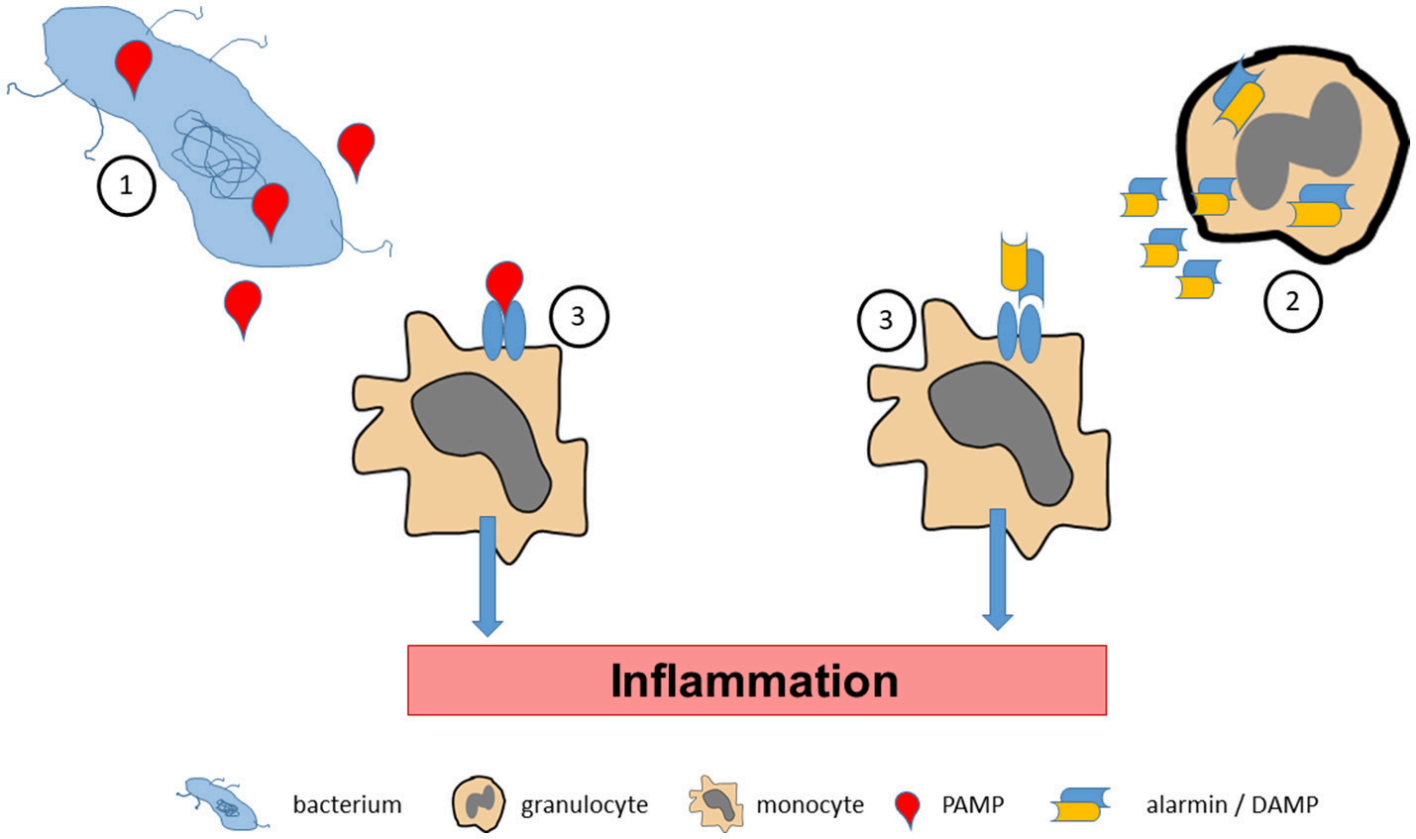
Pathogen associated molecular pattern molecules (PAMPs) are released by bacterial pathogens during infections (1). Accordingly, tissue stress or cellular activation may lead to release of endogenous alarmins or danger associated molecular pattern molecules (DAMPs) under sterile conditions (2). PAMPs and alarmins/DAMPs bind to and activate specific pattern recognition receptors on effector cells (3), which promote inflammatory processes.

It is now widely accepted that most pediatric rheumatic diseases are driven by mechanisms of both autoimmunity and auto-inflammation (1). It is believed that aberrant activation of dendritic cells (DC) due to presentation of autoantigens to T-cells in the context of pathological co-stimulation results in development of immune reactivity toward native antigens driven by autoreactive T- and B-cells. However, in many classical autoimmune diseases no dominant autoantigen has been identified so far and there is now increasing evidence that antigen-independent but self-directed inflammation, driven by local factors released during tissue damage or cellular stress, leads to activation of innate immune cells (2, 3). Primarily, inflammation is a protective response of the organism to infections or tissue damage eventually resulting in the elimination of the harmful trigger and tissue repair (4). However, rare inborn diseases of innate immunity, so-called auto-inflammatory diseases, demonstrated that uncontrolled activation of cytokine cascades, mutations leading to recurrent tissue or cell damage or aberrant receptors for detection of microbes may result in tissue specific recurrent inflammation (3). Cells of the innate immune system, first of all neutrophils and monocytes, recognize invading pathogens by so called pathogen associated molecular patterns (PAMPS), conserved structures specific for a distinct group of microorganisms, like LPS of gram-negative bacteria or single stranded RNA of viruses. These hallmarks of invading pathogens are recognized by conserved receptors on immune and non-immune cells called pattern recognition receptors (PRRs). PRRs include the toll like receptors (TLRs), which are highly conserved in different species and were first identified in drosophila, a species that does not possess an adaptive immune system (5). Upon binding of these PAMPS to specific receptors a transcriptional response is initiated resulting in the production of inflammatory cytokines like interleukin-1ß (IL-1ß), IL-6, or tumor necrosis factor (TNF) as well as of chemokines to promote the recruitment and activation of inflammatory cells at the site of infection/injury in order to combat pathogens and tissue damage (5). It is now clear that some PRRs, in addition to PAMPS, can also recognize own cellular molecules that are released during cell stress and tissue injury. In analog to PAMPs they are called DAMPs (damage or danger associated molecular patterns) or alarmins (Figure 1). Most alarmins are primarily intracellular molecules involved in different cellular processes. After release during cell damage or secretion by activated cells they act as extracellular danger signals. Like PAMPs, alarmins are recognized by PRRs (6). Interestingly, some receptors such as the TLR4 seem to be able to recognize PAMPs as well as alarmins (2, 7) Like PAMPS the recognition of alarmins induces a transcriptional response resulting in strong local sterile inflammation (8). Under physiological conditions the function of this inflammatory program is the induction of a tissue repair/remodeling situation resulting in reconstitution of the integrity of the organism. Alarmins include amongst others heat shock proteins (HSP60, 70, Gp96), high mobility group box 1 protein (HMGB1) but also S100-proteins (S100A8/S100A9 and S100A12,) which are the main topic of this review (8). In addition to proteins also lipoproteins and fatty acids, proteoglycans as well as nucleic acids can serve as alarmins.

Characteristic for all alarmins is the recognition by conserved receptors. S100-proteins for example are recognized by the TLR4/MD2 complex, while heat shock proteins are recognized by TLR2. As a functional consequence the expression of the receptors on different cell types also restrict their biological activity. Both TLR4 and TLR2 are expressed extracellularly, while dsDNA, ssRNA or DNA-immune complexes are sensed by intracellular receptors like TLR7 and TLR9 (9). Depending on the structure of the alarmin the binding to the receptor results in a downstream response similar to the binding of a pathogen. Alarmins are important signals to induce tissue repair mechanisms. However, there is current evidence, that excessive or inappropriate amounts and locations of alarmins can induce harm to tissue as well. High amounts of HMGB1 and HSP 70 for example have been identified in the synovia of rheumatoid arthritis patients (10, 11), dsDNA complexes induce interferon signaling in dendritic cells (12) and even serve as diagnostic criteria of systemic lupus erythematosus (SLE). In addition, incubation of healthy tissue or cells with alarmins can induce a sterile inflammation (13), while specific deletion of alarmins in genetically targeted mice results in amelioration of experimental disease like arthritis or sepsis. Thus, the whole system of alarmins as an inducer of cell injury on the one side and of tissue repair on the other side needs to be tightly controlled.

This suggests that alarmins as well as their binding partners (TLRs) can serve as therapeutic targets in certain disease settings. An example of this is again SLE, in which the drug chloroquine alters the pH of lysosomes, where TLR7 and 9 are located and thereby reduces the binding affinity of ds-DNA immune complexes to TLR9 and downregulates interferon production (9). The use of chloroquine has now come to a renaissance and is recommended for the basic treatment of SLE and used in nearly every patient (14).

## The Family of S100-Proteins

The most abundant alarmins in many inflammatory disorders, S100A8 and S100A9, belong to the group of S100-proteins defining a family of small (molecular weight of about 10–12 kDa) calcium-binding molecules. Members of this S100-protein family are characterized by a tissue or cell type-specific expression pattern. All S100-proteins have two calcium-binding sites of the so-called EF-hand type (15–17). Binding of calcium to these calcium binding sites induces conformational changes resulting in interaction of S100-proteins with different ligands or binding to specific receptors. A typical characteristic of most S100-proteins is the formation of homodimers, heterodimers and/or higher oligomers. Intracellularly S100-proteins have been described to be involved in many processes including cell cycle control, proliferation, differentiation, migration, metabolism, cellular dynamics, signaling, and cell death. A close correlation between high expression and release of different S100 proteins with disease activity has been shown in many inflammatory diseases, e.g., rheumatoid arthritis (RA), inflammatory bowel or lung disease, but also in Alzheimer's disease, cardiovascular disease and cancer (2, 15).

## S100A8 and S100A9: Major Calcium-Binding Proteins in Monocytes and Granulocytes

As mentioned above the most abundant alarmins in many clinically relevant diseases are S100A8 and S100A9. Both proteins have been initially described as myeloid-related protein 8 (MRP8) and MRP14 due to the fact that both molecules are expressed in high amounts in neutrophilic granulocytes and inflammatory monocytes/macrophages whereas they cannot be found in lymphocytes or resting tissue macrophages. In granulocytes S100A8/S100A9 represent more than 40% of the detergent soluble protein amount, in monocytes up to 5% (18). Synonyms of S100A8 and S100A9 are calgranulin A and calgranulin B, respectively, and complexes of both molecules have been also described as calprotectin. In addition, expression of both proteins is induced in some epithelial cells of gut and skin, osteoclasts and synoviocytes during inflammatory processes (7, 19).

Like many alarmins S100A8 and S100A9 exhibit primarily intracellular functions. Critical for biological functions of these proteins is formation of non-covalently associated hetero-complexes. S100A8/S100A9 heterodimers represent the structural basis of these proteins, monomers are not stable and homodimers seem not to play a relevant role in humans. With increasing calcium concentrations two S100A8/S100A9 dimers associate to (S100A8/S100A9)_2_ hetero-tetramers (20). Complexes of S100A8/S100A9 have been described to modulate cytoskeleton-membrane interactions in a calcium-dependent manner, which seems to be of relevance for cellular dynamics and migration of phagocytes. The latter effect seems to be mediated by regulating activity of small GTPases and polymerization of microtubules. The effect on tubulin polymerization is controlled by phosphorylation of S100A9 on threonine 113 by p38 mitogen-activated protein kinase (MAPK) (21). However, the intracellular functions of S100A8 and S100A9 are not well-defined.

## Secretion and Extracellular Effects of S100A8/S100A9

Beside the intracellular effects described above, S100A8 and S100A9 are secreted during many inflammatory diseases triggering inflammatory functions in many cell types, for example, endothelial cells, phagocytes, lymphocytes, or osteoclasts (22). S100A8 and S100A9 have been ascribed several extracellular functions, but the mode of secretion is still not completely clear since both proteins lack the necessary leader sequences for transport and release of the classical pathway via endoplasmic reticulum and Golgi complex. One potential mechanism is the passive release of both proteins due to necrosis of neutrophils and monocytes during inflammatory processes or during the formation of neutrophil extracellular traps (22). However, concentrations of these S100-proteins in sera do not correlate well with parameters of cell death. There is also a specific and energy-dependent release of S100A8/S100A9 by human monocytes which is induced within minutes after activation of these cells and which depends on activation of protein kinase C. Inhibitors of vesicular traffic through the endoplasmic reticulum and Golgi complex do not block release of S100A8/S100A9. The same is true for inhibitors of protein translation indicating that preformed S100-proteins are released during phagocyte activation. However, secretion of S100A8/S100A9 is an energy-dependent process and is significantly reduced by inhibitors of cellular energy metabolism and oxidative phosphorylation confirming an active and specific release pathway for these proteins (23). After induction of secretion S100A8/S100A9 complexes co-localize with microtubules and colchicine inhibits release of these molecules. Thus, the process of release is clearly distinct from classical secretion (eg., TNF) but shows also differences to the alternative pathway of release of IL-1ß (23). *In vivo* interaction of E-selectin with P-selectin glycoprotein ligand 1 (PSGL-1) triggers release of S100A8/S100A9 during rolling of neutrophils on TNF activated endothelial cells. Subsequently, S100A8/S100A9 act as an autocrine player promoting leukocyte adhesion to endothelium and transmigration. This process involves rapid ß2 integrin activation in a GTPase-dependent manner which results in reduced leukocyte rolling velocity and increased adhesion (24). Additional inflammatory stimuli of S100A8/S100A9 release in neutrophils include C5a, N-Formylmethionyl-leucyl-phenylalanine (fMLP), or monosodium urate crystals in a tyrosine kinase (Src)/spleen tyrosine kinase (Syk)- and tubulin-dependent manner (25).

S100A8/S100A9 acts on different cell types and induces several molecular pathways highly relevant in the pathology of arthritis. On endothelial cells S100A8/S100A9 induce an inflammatory response resulting in induction of cytokines, loss of cell-cell contacts, and increasing permeability of endothelial monolayers (26). Recently, we performed a genome-wide expression analysis with S100A8-stimulated monocytes. This analysis identified around 500 up- and 1,000 downregulated genes that were overrepresented in specific functional clusters, such as immune cell activation, cell migration, leukocyte activation, and signal transduction (NF-κB signaling) (27). S100A8/S100A9 induces expression of cytokines like TNF and IL-6, chemokines like CXCL-10 as well as matrix metallo-proteinases MMP3, MMP9, and particularly MMP13 which is involved in cartilage and bone metabolism (27, 28).

However, continuous stimulation of TLR4 with S100A8/S100A9 may induce a status of “tolerance” in phagocytes as described for PAMPS like LPS and alarmins like heat shock proteins or HMGB1 (29–31). In addition, prolonged exposure of myeloid progenitor cells modulates development of dendritic cells and so called myeloid derived suppressor cells depending on time and dose of S100-stimulus (32).

S100A8/ S100A9 has an anti-apoptotic effect on neutrophils and increases cell survival, a pathway involving TLR4, CD11b/CD18, and mitogen activated protein kinase signaling (33).

## Receptors for S100A8/S100A9

After release into the extracellular compartment S100A8/S100A9 molecules are enriched at sites of inflammation by binding glycosaminoglycans. In addition, S100A8/S100A9 may interact with specific receptors, TLR4 and the receptor for advanced glycation end products (RAGE) (34). Another receptor, EMMPRIN (synonyms BASIGIN and CD149), binds S100A9 and has been reported to trigger monocyte/macrophage migration. However, the physiological relevance of this receptor for S100-biology is yet not clear (35).

Although S100A8/S100A9 bind to RAGE and especially carboxylated N-glycans expressed on this receptor knock-out of RAGE in myeloid cells does not interfere with the inflammatory response induced by S100A8/S100A9. In contrast, knock-out of TLR4 in murine phagocytes completely abolishes the response of these cells toward S100A8/S100A9 stimulation (36). The relevance of S100A8/S100A9-mediated TLR4 signaling was confirmed in human monocytes demonstrating an almost identical expression pattern induced by the classical TLR4-ligand LPS and S100A8 (27). Accordingly, transfection of HEK cells with TLR4 induces S100-sensitivity of these cells whereas RAGE transfection has no effect. S100A8/S100A9-binding to TLR4 activates MyD88 and TRIF-dependent signaling and results in activation of NF-kB and induction of inflammatory gene expression (27, 36).

Since TLR4 is also the LPS receptor possible endotoxin contamination of purified S100A8/S100A9 could be a major bias regarding inflammatory effects of these proteins. However, this possibility was excluded in several independent approaches. First of all, knock-out of S100A9 in mice has an anti-inflammatory effect in many murine models even under sterile conditions in the absence of any microbial stimulus (7, 22, 28). Furthermore, LPS contaminations of S100 charges were excluded by Limulus assay. Blocking LPS by the endotoxin antagonist polymyxin B has no effect on S100A8/S100A9 activities. Heating of S100A8/S100A9 samples on the other hand completely abolished inflammatory activities of these proteins under conditions which have no influence on LPS activity in the same experiments (27, 36). Last but not least, we have recently identified the specific TLR4-binding site within the S100A8 and S100A9 molecules which were confirmed by targeted mutagenesis, peptide binding and structural analysis. Point mutations in the TLR4-binding site of S100A8 or S100A9 abolished inflammatory activity which is the final proof to exclude any effect by LPS contaminations (37).

**Figure 2:**
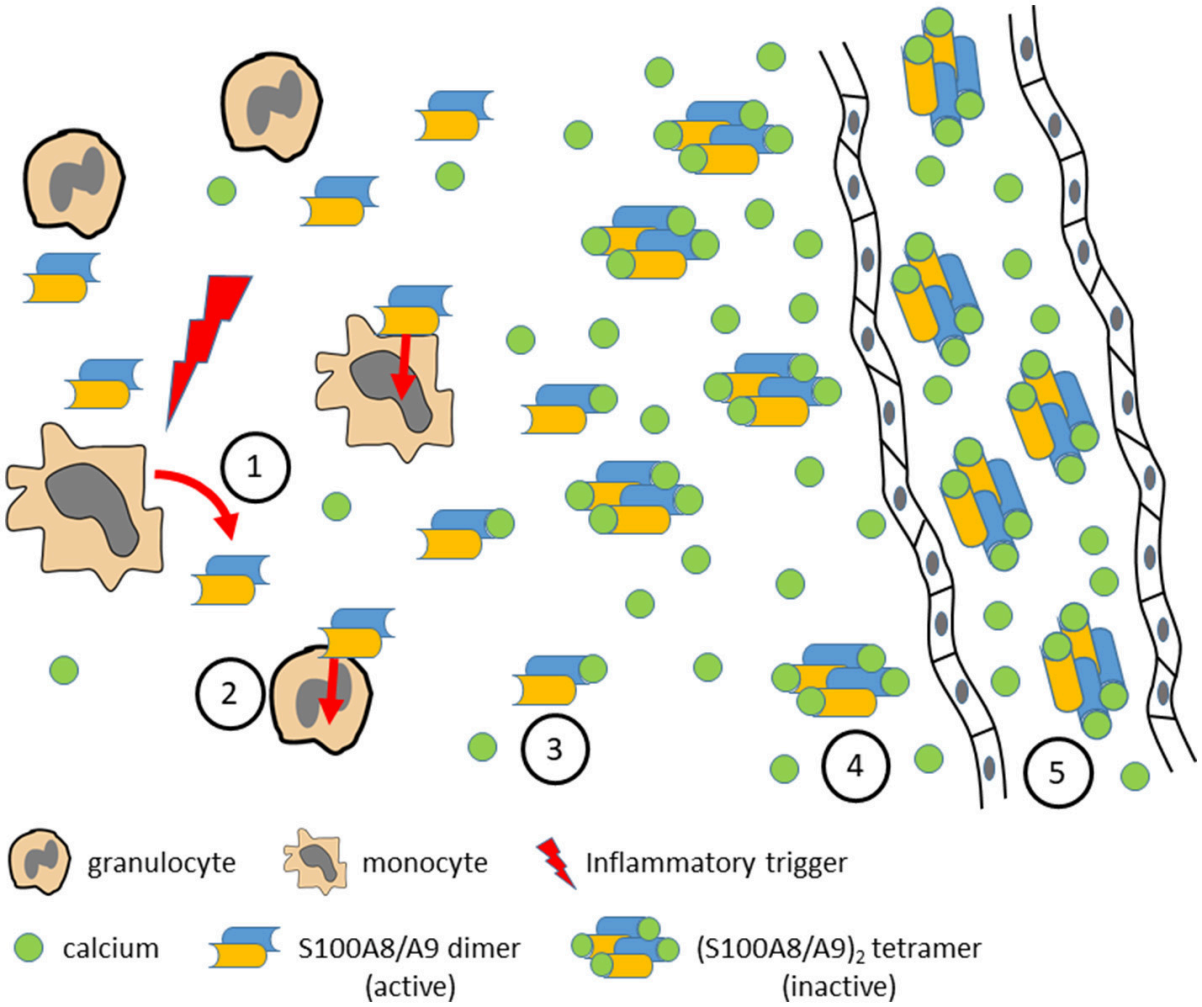
Various inflammatory triggers lead to local release of S100A8/S100A9 dimers by activated monocytes or granulocytes (1). S100A8/S100A9 may bind to TLR4 on different target cells and amplify and perpetuate the inflammatory response (2). With increasing calcium concentrations toward systemic circulation S100A8/S100A9 dimers bind calcium ions (3) and form (S100A8/S100A9)_2_ tetramers (4) which show no inflammatory activity any more due to the fact that the TLR4 binding site is hidden in the tetramer interface. In systemic circulation high calcium concentrations prevent systemic inflammatory side effects by stabilization of inactive (S100A8/S100A9)_2_ tetramers (5) which, however, are useful biomarkers for monitoring local disease activity.

Recently we unraveled a novel regulatory mechanism which restricts the inflammatory effects of S100A8/S100A9 to the local process of inflammation. Interaction of S100A8/S100A9 with TLR4/MD2 is mediated by peptide sequences of about 10–15 amino acids within the second calcium-binding EF-hands of both S100-subunits. These TLR4 binding structures are freely accessible in heterodimers of S100A8/S100A9 which are released during inflammation by monocytes, macrophages and granulocytes. In the presence of high extracellular calcium concentrations S100A8/S100A9 dimers associate to (S100A8/S100A9)_2_ tetramers which hide the specific TLR4/MD2-binding peptides within the tetramer interphase, thus representing an auto-inhibitory process limiting S100-effects to local sites of inflammation and avoiding undesirable systemic effects (Figure 2). Loss of this auto-inhibitory mechanism results in fatal inflammation in an animal model of TNF-driven arthritis and psoriasis (37).

## S100A8/A9 in Arthritis and Autoimmunity

S100A8 and S100A9 were initially identified as the two major calcium-binding proteins highly expressed in inflammatory granulocytes and macrophages during RA (38). Synovitis during RA is characterized by a high abundance of neutrophils and macrophages expressing S100A8 and S100A9 especially at the cartilage–pannus junctions indicating that S100-expression is closely associated with cartilage destruction and bone erosion in arthritis. Concentrations of S100A8/S100A9 are very high at the side of inflammation, ie., the synovial fluid, and correlate well with serum concentrations and disease severity in patients with RA (39). Also, in psoriasis arthritis patients, expression of S100A8 andS100A9 is very high in the synovial sub-lining layer and particularly in perivascular areas (40).

Functional relevance of S100A8/S100A9 expression was further confirmed by analysis of experimental models of arthritis and synovial inflammation in S100A9^−/−^ mice. Lack of S100A8/A9 expression decreases joint inflammation, protease expression and cartilage destruction during antigen-induced arthritis (28). In this context S100A8 induces activating Fcγ receptors I and IV in macrophages in inflamed synovium in a TLR-4 dependent manner which eventually results in bone erosion (41, 42). There is a synergetic effect between TNF, IL-1ß, IL-17, and S100A8 leading to induction of erosive MMPs and exaggeration of cartilage erosion during arthritis (43). In addition, S100A8 and S100A9 have been shown to promote the development of functional autoreactive CD8+ T-cells in a mouse model of CD40L-induced lupus-like disease (44).

## Expression of S100A12 in Inflammatory Processes

The third S100-protein expressed in granulocytes and monocytes is S100A12. Expression of S100A12 correlates closely with S100A8/S100A9, however, in at least 10-fold lower quantities. S100A12 resulted from a duplication of the S100A9 gene during evolution (15, 45). However, expression levels obviously decreased significantly compared to S100A9 during evolution and the gene is disrupted and no S100A12 protein is found in rodents including mice. Biological activity of S100A12 is low compared to S100A8/S100A9. There is no clear evidence *in vivo* whether S100A12 has a relevant function in inflammatory processes. However, expression of this protein correlates very well with disease activity in many pathological conditions and S100A12 is a useful biomarker for monitoring disease activity in many clinically relevant disorders like rheumatoid arthritis, inflammatory bowel disease, vasculitis, or psoriasis (46–48).

## S100 Proteins as Biomarkers of Inflammation

Since alarmins and cytokines are essential part of the pathophysiology of pediatric rheumatic diseases they are candidates for biomarkers of inflammatory processes. However, to be useful biomarkers should be able to support initial diagnosis, reflect disease activity, and predict the further outcome of inflammatory diseases with high diagnostic accuracy. Feasible biomarkers should be obtained through non-invasive, easily performed, reproducible, and cost-effective procedures.

S100 proteins are part of the local inflammatory process and reflect the disease activity when measured in the serum. Phagocyte-specific S100-proteins have been established as useful markers of both local and systemic inflammation. They correlate with disease activity in rheumatic diseases, vasculitis, inflammatory bowel disease, pulmonary diseases, and infections (46–58). Their stability makes these proteins useful as biomarkers for the monitoring of pediatric rheumatic diseases in clinical practice. S100 proteins are stable at room temperature for several days in separated serum, so serum samples can be sent at room temperature (59). Additionally, commercial assays are available especially for the detection of S100A8/S100A9 but only some of these assays are certified for use in clinical diagnostics. However, these assays are not strictly comparable and especially the concentrations vary among different providers. Therefore, a strict evaluation including validation and standardization of these assays is mandatory before introducing commercial assays in clinical practice. For some of these assays validation studies have been already performed and some assays can be recommended for use to confirm inactive disease in clinical practice (60). However, all available assays have a limited linearity in serum compared to standard buffer at higher concentrations often requiring serial dilutions of individual samples. Serum concentrations of S100-proteins are independent of age and gender (59). Normalization of S100 levels can take 8 [in CAPS with effective canakinumab treatment (61)] to 30 days [in SJIA with effective anakinra treatment (62)].

## S100 Proteins in Pediatric Rheumatic Diseases

### Juvenile Idiopathic Arthritis (JIA)

#### Pathophysiology and Marker of Disease Activity

Over the last 20 years the importance of S100-proteins as biomarkers of inflammatory activity and the understanding of its pathogenetic role in JIA has evolved rapidly. In 2000, Frosch et al. could demonstrate that S100A8 and S100A9 are specifically released during interaction of activated monocytes with TNF-stimulated endothelial cells. In JIA patients, S100A8, and S100A9 were strongly expressed in infiltrating neutrophils and monocytes within the inflamed joints and could be found in significantly higher concentrations in synovial fluid compared with serum. After intraarticular triamcinolone therapy, the serum concentrations of S100A8/S100A9 decreased significantly in the serum of therapy responders, whereas no differences were found in patients who showed no clinical benefit (63).

Comparable results could be obtained for S100A12. S100A12 serum concentrations were determined in 124 patients with chronic active polyarticular, oligoarticular, or systemic-onset JIA (SJIA). The mean serum level of S100A12 was 395 ng/ml in patients with active polyarticular JRA and 325 ng/ml in patients with active oligoarticular JIA (normal < 120 ng/ml). The level of S100A12 was ~ 10-fold higher in synovial fluid than in serum, indicating release at sites of local inflammation. Notably, in patients with SJIA, the mean level of S100A12 was 3700 ng/ml. Moreover, serum levels decreased in response to different anti-inflammatory therapies (i.e., intraarticular injections of corticosteroids, methotrexate (MTX), or etanercept). Moreover, S100A12 levels were elevated in 20 patients who experienced disease flares after the initial induction of remission, even weeks before the relapses became clinically apparent. This finding demonstrated that S100-proteins might indicate synovial inflammation even when other signs of arthritis are absent (52).

#### Marker of Subclinical Inflammation

Accordingly, the potential of S100-proteins to indicate subclinical inflammation was evaluated in a large prospective, open, multicenter, medication-withdrawal randomized clinical trial including 364 patients. This study aimed to analyze whether longer MTX treatment during remission of JIA prevents flares after withdrawal of medication and whether specific biomarkers identify patients at risk for flares. Primary outcome was relapse rate in the 2 treatment groups (withdrawal after 6 or 12 months); secondary outcome was time to relapse. Besides the finding that in patients with JIA in remission, a 12- vs. 6-month withdrawal of MTX did not reduce the relapse rate, it could be demonstrated that higher S100A8/S100A9 concentrations at time of MTX withdrawal were associated with risk of relapse after discontinuing MTX (59).

Clinical inactive disease with elevated inflammatory markers can be defined as subclinical disease activity, which may result in unstable remission (i.e., a status of clinical but not immunological remission). Therefore, in a sub-analysis of this study S100A12, S100A8/S100A9 as well as the acute phase reactant high-sensitivity C reactive protein (hsCRP) were compared as predictive biomarkers for the risk of a flare within a time frame of 6 months. Clinical or standard laboratory parameters could not differentiate between patients at risk of relapse and those not at risk. On the other hand S100A12 and S100A8/S100A9 levels were significantly higher in patients who subsequently developed flares than in patients with stable remission (64).

To implement these biomarkers for further studies and use in clinical practice the performance of different enzyme-linked immunosorbent assays (ELISAs) in order to validate systems available for routine use were tested. The tested commercial S100A8/S100A9 and S100A12 ELISAs showed a performance comparable to well-established experimental ELISA protocols when assay-specific cutoffs for the indication of relapse prediction were thoroughly applied (60). In another study S100A8/S100A9 levels before discontinuation of anti-tumor necrosis factor (TNF)-inhibitors were analyzed retrospectively. Patients who flared within 6 months after treatment discontinuation had higher S100A8/S100A9 levels compared to patients with stable remission. Results were confirmed by a commercial ELISA assay with high reproducibility but different overall levels (65). As mentioned above analysis of higher serum concentrations may require serial dilutions of individual samples to obtain reliable results. A recent study analyzed the relationship between serum S100A8/S100A9 and S100A12 and the maintenance of clinical inactive disease (CID) in patients with polyarticular forms of juvenile idiopathic arthritis (PF-JIA) while on anti-TNF- therapy and disease flare following withdrawal of treatment. Here, serum S100 levels did not predict maintenance of CID or disease flare, with S100A12 levels only moderately correlating inversely with time to disease flare (66). Further studies are needed to evaluate the clinical use of S100-proteins for stopping treatment.

#### Marker of Response to Therapy

Besides supporting tools for stopping treatment in remission there is an unmet need for biomarkers with which to identify patients who will respond well to anti-inflammatory therapy. Around one-third of patients with juvenile idiopathic arthritis (JIA) fail to respond to first-line MTX or TNF therapy, with even fewer achieving ≥ American College of Rheumatology Pediatric 70% criteria for response (ACRpedi70). Within the Childhood Arthritis Response to Medication Study (CHARMS) the prognostic value of baseline serum proteins (S100A8/S100A9, inflammatory cytokines, CRP), ESR and clinical variables in response to MTX was analyzed to identify whether the patient is likely to respond well to MTX. High disease activity (high serum S100A8/S100A9, active joint count, or physician's score) pre-MTX was observed in a subgroup of patients with a better response to therapy. In a multivariable analysis, after accounting for S100A8/S100A9 at baseline, no other factors were independently significantly associated with outcome (67). High levels of baseline S100A8/S100A9 are associated with good response to anti-TNF treatment. Baseline S100A8/S100A9 levels in patients before treatment with TNF-inhibitors were higher in responders compared to non-responders. Levels decreased after start of treatment only in responders. Change in JADAS-10 was correlated with baseline S100A8/S100A9 levels and documented the correlation of S100A8/S100A9 with disease activity (65). These results could also be confirmed for S100A12. Responders to MTX or anti-TNF treatment can be identified by higher pretreatment S100A12 serum concentration levels and baseline serum S100A12 in both univariate and multivariate regression models was significantly associated with change in JADAS-10 (68).

### Juvenile Dermatomyositis (JDM)

The aetiopathogenesis of JDM and in particular the contribution of monocytes or macrophages, which are frequently observed to infiltrate muscle tissue very early in the disease process, remains poorly understood. Early results indicated a clear association of expression of S100A8 and S100A9 by infiltrating macrophages with degeneration of myofibers in muscle biopsies of patients with dermatomyositis, polymyositis, and inclusion body myositis. Furthermore, S100A8/S100A9 complex inhibited proliferation and differentiation of myoblasts and induced apoptosis via activation of caspase-3. In the course of inflammatory myopathies, activated macrophages seem to promote destruction and impair regeneration of myocytes via secretion of S100A8/S100A9 (69). These findings could be confirmed in a follow-up study. Here, S100A8/S100A9 levels of 56 JDM patients were compared with clinical measures of disease activity. S100A8/S100A9 serum levels correlated with physician's global assessment of disease activity in JDM and muscle strength/endurance, childhood myositis assessment score. S100A8/S100A9 was widely expressed by CD68+ macrophages in JDM muscle tissue. When cultured with human myoblasts, S100A8 led to the secretion of MCP-1 and IL-6, which was enhanced by ER stress. However, the usefulness of serum S100A8/S100A9 as a potential biomarker for disease activity in JDM has to be confirmed in further studies (70).

### Autoinflammatory Diseases

Markedly elevated S100 levels are a hallmark of SJIA, Familial Mediterranean Fever (FMF) and PSTPIP1 associated inflammatory diseases (PAID) such as pyogenic sterile arthritis, pyoderma gangrenosum, and acne (PAPA) syndrome or PSTPIP1-associated myeloid-related proteinaemia inflammatory (PAMI) syndrome (71). Here, S100 levels can differentiate these conditions from other infectious or auto-inflammatory conditions (Table 1). Hypersecretion of S100 proteins in these diseases can result in a sterile inflammatory environment, which triggers pro-inflammatory cytokine as well as further S100A8/S100A9 and S100A12 expression and thus can perpetuate disease activity (72, 73). In contrast, in the cryopyrin associated periodic syndromes (CAPS) or periodic fever, aphthous stomatitis, pharyngitis, adenitis (PFAPA) syndrome S100 levels are lower and within the range of other inflammatory diseases and cannot be used to differentiate from infectious diseases; however, they do correlate with disease activity.

**Table 1:** Serum concentration of phagocyte-specific S100 proteins in inflammatory diseases [adapted and updated from Kessel et al. (73)].

	**S100A8/A9 levels (ng/ml)**	**N^†^**	**References**	**S100A12 levels (ng/ml)**	**N^†^**	**References**
**POLYGENIC AUTOINFLAMMATORY DISEASES**						
*Systemic-onset JIA*	14,920 ± 4,030	60	(76)	7,190 ± 2,690	60	(75)
	24,750 ± 11,410	20	(77)	3,700 (1,080)^**^	33	(78)
*Polyarthritis JIA*	2,380 ± 530	89	(52, 63)	395 (45)^**^	89	(78)
*PFAPA*	3846 ± 1197	15	(79)	685 ± 210	15	(79)
**MONOGENIC AUTOINFLAMMATORY DISEASES**						
*FMF*	110,000 ± 82,000	20	(71)	6,720 ± 4,960	17	(75)
				33,500 (22,200)^**^	7	(56)
*PAPA*	116,000 ± 74,000	11	(71)	–		
*PAMI*	2,045,000 ± 1,300,000	13	(71)	–		
*NOMID*	2,830 ± 580	18	(76)	720 ± 450	18	(75)
*MWS*	4,390 (2535)^*^	12	(61)	150 ± 60	17	(75)
*FCAS*	3,600 (4610)^*^	5	(61)	–	–	–
**INFECTIONS**						
*Severe febrile infections*	3,720 ± 870	66	(76)	470 ± 160	83	(75)
Healthy controls	340 ± 70	50	(76)	50 ± 10	45	(75)
				50 (5)^**^	74	(50)

FCAS, Familial cold autoinflammatory syndrome, FMF, familial Mediterranean fever; JIA, juvenile idiopathic arthritis; MWS, Muckle Wells syndrome; NOMID, Neonatal onset multisystem inflammatory disorder; PAMI, PSTPIP1-associated myeloid-related proteinemia inflammatory; PAPA, pyogenic sterile arthritis, pyoderma gangrenosum, and acne syndrome; PFAPA, periodic fever, aphthous stomatitis, pharyngitis, adenitis syndrome [

* mean (standard deviation),

** mean (standard error of the mean), all other data are mean ± 95% confidence interval,

† *N, number of patients studied]*.

## Monogenic Auto-Inflammatory Syndromes

### Familial Mediterranean Fever (FMF)

#### Pathophysiology

FMF is an auto-inflammatory syndrome associated with the activation of phagocytic cells and an over-secretion of IL-1β. The discovery of pyrin mutations as the genetic basis of this auto-inflammatory disorder identified the dysfunction of intracellular processes, e.g., alternative secretory pathways, and immune dysregulation involving inflammasome-dependent recruitment and processing of IL-1β as causes of FMF (74). During inflammatory attacks of FMF serum levels of S100A8/S100A9 and S100A12 are massively elevated and significantly higher than in patients with CAPS (75). Both S100A8/S100A9 and S100A12 exhibit pro-inflammatory effects *in vitro* at concentration found in FMF patients *in vivo* during active disease (Table 1). This hypothesis is further supported by the observation that S100A8/S100A9 co-localizes with the cytoskeleton and a Golgi-independent but tubulin-dependent release has been shown (21, 23). Pyrin is likewise associated with these structures while colchicine blocks tubulin-dependent processes at the molecular level and is therefore a possible inhibitor of alternative secretion of S100 proteins (80).

#### Marker of Disease Activity

During acute attacks serum levels of S100A8/S100A9 and S100A12 are massively elevated (71, 72). Moreover, patients with FMF well-controlled with anti-inflammatory treatment have significantly decreased serum levels (72). S100A12 may also allow stratification of FMF patients according to disease severity (72). Overall, S100A12 levels show an excellent correlation to disease activity (56, 75). S100A12 serum levels in patients with unstable disease under colchicine treatment were significantly higher than those without inflammatory attacks, supposed as stable disease. Moreover, homozygous MEFV mutation carriers exhibited clearly increased S100A12 serum levels despite of no clinical disease activity while classical inflammatory markers were in the range of normal controls. Also, heterozygous MEFV mutation carriers have significantly elevated S100A12 serum levels while classical inflammation markers were not increased (56). These findings indicate ongoing subclinical inflammatory activity of the innate immune system in otherwise clinically stable individuals.

### PSTPIP1 Associated Inflammatory Diseases (PAID)

PAPA syndrome seems to be only one clinical entity within the expanding spectrum of PAID caused by mutations in PSTPIP1 (81). PAMI syndrome (PSTPIP1 E250K mutation) is a PAID presenting with clinical and biochemical features not found in patients with classical PAPA syndrome (71). Mutated PSTPIP1 markedly increases pyrin binding and IL-1ß production by peripheral blood leukocytes from patients with PAPA and in cell lines transfected with both PAPA associated mutants (82). Moreover, PAPA-associated PSTPIP1 mutants activate pyrin, thereby allowing it to interact with ASC and facilitate ASC oligomerization into an active ASC pyroptosome (83). A hallmark of PAID are very high (PAPA: 116 ± 74 μg/ml) or massively elevated S100A8/S100A9 serum concentrations (PAMI: 2,070 ± 1,190 μg/ml vs. 0.48±0.1 μg/ml in healthy controls) (71). Although the exact role of S100A8/S100A9 in the pathogenesis of PAID is not yet clear, there are important links between S100A8/S100A9, pyrin and PSTPIP1. S100A8/S100A9 serum levels are also highly elevated in FMF (110 ± 82 μg/ml) (71). Like PSTPIP1 and pyrin, S100A8, and S100A9 are highly expressed in phagocytes. Both proteins bind to both the subcellular actin network and microtubules in a calcium dependent manner (21). Interestingly, IL-1ß secretion is only apparent in monocytes of PAPA patients after stimulation with the exogenous TLR-4 ligand LPS (84), which points to a putative role of endogenous TLR-4 ligands S100A8 and S100A9 for the release of IL-1ß from PAPA monocytes.

### Cryopyrin-Associated Periodic Syndromes (CAPS)

#### Pathophysiology

Cryopyrin-associated periodic syndromes (CAPS) comprise a group of rare auto-inflammatory diseases, which include the Familial Cold Auto-inflammatory Syndrome (FCAS), the Muckle-Wells Syndrome (MWS), and the Neonatal-Onset Multiorgan Inflammatory Disease (NOMID) and are caused by mutations in the *NLRP3* (*CIAS1/NALP3/PYPAF1)* gene, encoding for cryopyrin/NALP3 protein. Cryopyrin controls the assembly of proteins into the inflammasome complex, which regulates caspase-1 activity that induces the conversion of pro-IL-1β to biologically active IL-1β (85–88). Uncontrolled pro-IL-1β processing results in a constitutive excess of IL-1β release from phagocytic cells of CAPS patients (89–91). IL-1 hypersecretion is not easy to determine *in vivo* and is only one factor among others involved in a complex immune dysregulation including phagocyte activation during auto-inflammation (76). Although the exact role of the S100-proteins in CAPS has not yet been fully understood, they seem to reflect IL-1ß-driven inflammation in CAPS.

#### Marker of Disease Activity

Various states of subclinical disease activity were demonstrated in all categories of CAPS, depending on the type of anti-IL-1 therapy. Here, S100 levels were compared with CRP and ESR and seemed to have a higher sensitivity to detect subclinical inflammation. In this context, S100A8/S100A9 proved to be a sensitive biomarker for monitoring disease activity, and response to IL-1 blockade in patients with CAPS and also indicated subclinical inflammation when CRP and ESR were already normal (57, 61). S100A12 has been shown to be elevated in patients with active NOMID and MWS (75). In patients with CAPS treated with IL-1-blockers, S100A12 and S100A8/S100A9 correlate with inflammatory activity and decline rapidly along with a normalization of neutrophil counts (92).

## Polygenic Auto-Inflammatory Diseases

### Systemic Juvenile Idiopathic Arthritis (SJIA)

#### Pathophysiology

SJIA is a severe systemic inflammatory disease in childhood with significant morbidity and serious complications, especially in those children with a therapy-resistant course. Although defined as a subtype of JIA, the disease nowadays is attributed to the auto-inflammatory syndromes with a significant role of IL-1. Thus, clinical symptoms can be assigned to dysregulated innate immune mechanisms with only little involvement of adaptive immunity. Serum of SJIA patients induces the transcription of genes of the innate immune system including IL-1 in peripheral blood mononuclear cells (PBMCs) and activated monocytes from patients with SJIA secrete significantly higher amounts of IL-1β in comparison with monocytes of healthy controls (93) The predominant role of the innate immune system is furthermore underscored by very high S100A8/S100A9 and S100A12 serum levels (75, 76). The hypersecretion of IL-1, IL-18, S100A8/S100A9, and S100A12 indicates an important aspect regarding the pathogenesis of SJIA since they are all released by the alternative secretory pathway. In contrast to IL-1 and IL-18, S100-proteins are not processed by caspase 1 prior to release (23). Thus, a loss of control of the alternative secretory pathway downstream of caspase 1 has been proposed to be involved in release of pro-inflammatory proteins leading to the inflammatory process of SJIA (94). However, it cannot be determined whether secretion of IL-1β, IL-6, or S100-proteins is a primary or secondary step in the cause-and-effect chain of SJIA (95).

#### Detection Marker in Fever of Unknown Origin

At initial presentation, SJIA is difficult to differentiate from severe systemic infections. S100A8/S100A9 serum levels are closely correlated to disease activity in SJIA and these high concentrations can be found neither in other forms of inflammatory arthritis, nor in other autoimmune or infectious diseases (52, 78)- in contrast to markers like CRP, which are not able to differentiate SJIA from other causes of FUO (76, 96, 97). The same applies for S100A12 (75, 98). Although SJIA cannot be differentiated from FMF or PAID in this context, these autoinflammatory diseases may at least clinically be differentiated from other causes of FUO.

#### Marker of Disease Activity

S100A8/S100A9 serum levels correlate closely with response to drug treatment and disease activity and therefore might be an additional measurement for monitoring anti-inflammatory treatment of individual patients with SJIA (62, 77). S100A8/S100A9 serum concentrations are the first predictive biomarker in SJIA indicating subclinical disease activity and stratifying patients at risk of relapse during times of clinically inactive disease (77) and might be able to predict response to treatment with anakinra (62).

## Periodic Fever, Aphthous Stomatitis, Pharyngitis, Cervical Adenitis (PFAPA) Syndrome

PFAPA syndrome is characterized by fever flares accompanied by pharyngitis, adenitis, and/or aphthous stomatitis without evidence of infection, asymptomatic intervals between the flares, and onset before the age of 5 years (99). The pathogenic mechanism of this syndrome is not known, but it has been shown that IL-1ß production by monocytes is dysregulated in patients with PFAPA syndrome. Twenty percentage of enrolled patients were found to have NLRP3 variants, suggesting that inflammasome-related genes might be involved in this auto-inflammatory syndrome. S100A8/S100A9 and S100A12 are upregulated in flares but within the range of healthy control in symptom-free intervals. The levels of active patients are within those of systemic infections and have no additional diagnostic value in PFAPA (79).

## Outlook

There is now increasing evidence that innate immune mechanisms triggered by local tissue signals, so called alarmins or DAMPs, are crucial factors in the pathogenesis of many pediatric rheumatic diseases. S100-alarmins, especially S100A8/S100A9, are highly upregulated in different forms of arthritis and autoimmune diseases in children. Specific secretion of S100-alarmins at the local site of inflammation by activated phagocytes makes these molecules useful markers for monitoring disease activity. For early diagnosis of SJIA S100A8/S100A9 is currently the most specific biomarker and is used in several specialized centers. In addition, it is used in clinical studies for prediction of disease flares in patients with JIA or RA in clinical remission on medication after stopping or reduction of therapy. However, use in clinical routine is limited by the fact that no commercial assay is available which guaranties reliable data in the whole range of serum levels found in different inflammatory disorders. Novel approaches to follow expression of these molecules *in vivo* by molecular imaging techniques, which already work very well in preclinical models, may even improve the diagnostic value of these molecules. There is already a small molecular compound described which can be used for monitoring S100A8/S100A9 expression in preclinical models of inflammation *in vivo*. Since the structure of this tracer is based on Q-compounds already used in clinical trials with very low toxicity, transfer of such a tracer into clinical practice may be feasible within the next years (100, 101). Preclinical models in mice have also confirmed a functional role of S100A8/S100A9 in the process of arthritis and autoimmunity. Especially in some auto-inflammatory diseases like SJIA, FMF, and PAID S100-alarmins seem to play a dominant role. The Q-compounds mentioned above have been shown to specifically inhibit binding of S100A9 to TLR4 and RAGE (34). These drugs block the infiltration and activation of phagocytes in experimental models of inflammation (24) In addition, laquinimod, a member of the Q-compound family, showed significant effects in a randomized clinical trial (phase III) for treatment of relapsing–remitting multiple sclerosis (102) and in patients with Crohn's disease (phase II) (103). Treatment with laquinimod was well-tolerated and not associated with major side effects. There are no published data showing therapeutic effects of laquinimod or any other Q-compounds in pediatric rheumatic diseases so far. The recent identification of the TLR4-binding site in S100A8 and S100A9 offers novel molecular structures for targeted inhibition of these interaction. Since the active form of S100A8/S100A9 is restricted to local sites of inflammation such an approach may have very limited systemic side effects. However, data regarding blocking antibodies directed against these specific binding structures in preclinical mouse models are missing so far and would be a prerequisite for transfer of this novel therapeutic approach into clinical trials. In addition, pharmacological inhibition of the non-classical secretory pathway of S100A8/S100A9 may be an alternative strategy to specifically address this inflammatory mechanism. Interestingly, colchicine, which is already used for the treatment of FMF, has been shown to inhibit secretion of S100A8/S100A9 by activated phagocytes. Taken together there are now several lines of future research in the field of S100-biology which may offer innovative diagnostic or even therapeutic approaches for pediatric rheumatic diseases.

## Author Contributions

All authors listed have made a substantial, direct and intellectual contribution to the work, and approved it for publication.

### Conflict of Interest Statement

The authors declare that the research was conducted in the absence of any commercial or financial relationships that could be construed as a potential conflict of interest.
